# Low-frequency ultrasound-assisted preparation of taurine-loaded nanoemulsion and its preservative effect on fresh-cut pitaya

**DOI:** 10.3389/fnut.2026.1925794

**Published:** 2026-09-07

**Authors:** Yunpo Huang, Zheng Zhan, Xuejiao Ren, Chen Li

**Affiliations:** 1 College of Food and Health, Jinzhou Medical University, Jinzhou, China; 2 Liaoning Provincial Professional Technology Innovation Center of Meat Processing and Quality-Safety Control, Jinzhou, China

**Keywords:** fresh-cut pitaya, nanoemulsion, quality preservation, taurine, ultrasound

## Abstract

In this study, a taurine-loaded nanoemulsion was prepared using low-frequency ultrasound at 20 kHz for 20 min, and its preservative effects on fresh-cut pitaya was evaluated. The optimal preparation conditions were 400 W ultrasonic power and 1.0% taurine, which produced a nanoemulsion with a mean particle size of 112.58 nm, a polydispesity index of 0.17, a zeta potential of −35.84 mV, an encapsulation efficiency of 85.27%, and good stability for 28 days at 4 °C. Compared with control, free taurine, and blank nanoemulsion treatments, the taurine-loaded nanoemulsion (T-NE) significantly delayed quality deterioration during 8 days of storage at 4 °C. On day 8, the treated fruit showed a weight loss of 8.24%, a firmness loss of 5.18 N, maintained higher soluble solids (10.62 °Brix), a total phenolic content of 65.43 mg/100 g, and DPPH radical scavenging activity of 75.38%. The treatment also enhanced superoxide dismutase and catalase activity, inhibited polyphenol oxidase activity, lowered malondialdehyde accumulation (4.68 nmol/g), and reduced the total viable count and mold and yeast count to 4.73 log CFU/g and 3.65 log CFU/g, respectively. These findings indicate that the taurine-loaded nanoemulsion can prolong the refrigerated shelf life of fresh-cut pitaya and may have broader applications in fruit and vegetable preservation.

## 1 Introduction

Fresh-cut pitaya fruit is valued for its nutritional properties and convenience. However, cutting and subsequent storage accelerate water loss, browning, softening, and microbial contamination, thereby reducing product quality and shelf life. Fresh-cut pitaya typically remains marketable for only 3–5 days at 4 °C (1, 2). Traditional preservation methods, such as low-temperature storage and chemical preservative treatment, are effective; however, they have limitations, such as high costs, residual risks, and low consumer acceptance (3). Therefore, the development of safe and efficient preservation technologies remains an important priority in fresh-produce processing.

Taurine, or 2-aminoethanesulfonic acid, is a naturally occurring sulfur-containing amino acid with antioxidant, anti-browning, and osmoregulatory properties (4). Taurine can reduce oxidative stress and stabilize cellular membranes by supporting calcium homeostasis and maintaining mitochondrial membrane integrity (5, 6). These properties make taurine a potential preservative for fresh produce. However, due to its high water solubility, taurine is easily lost during direct spraying or soaking treatments, making it difficult to form a stable and long-lasting protective film on the surface of fruits and vegetables, thereby limiting its preservation effect (5, 6). Owing to their advantages, such as small particle size, large specific surface area, high stability, and good bioavailability, nanoliposomes can be used as efficient delivery systems for active ingredients (7). Embedding taurine in nanoliposomes not only enhances its adhesion and permeability on the surface of fruits but also enables its sustained release, thereby improving preservation efficiency. Recent studies have demonstrated that nanoemulsion-based delivery systems can effectively incorporate various bioactive compounds for postharvest preservation applications (8, 9).

Low-frequency ultrasound-assisted emulsification has attracted interest because it is relatively simple, energy efficient, and capable of producing small droplets with narrow size distributions (10, 11). Acoustic cavitation generated during ultrasonication creates intense shear forces and micro-turbulence that break up large droplets into nanoscale dimensions. A few studies have reported the preparation of active compound-loaded nanoemulsions using low-frequency ultrasound and their application in fresh-cut fruit and vegetable preservation (12–14). Therefore, this study aimed to prepare a taurine-loaded nanoemulsion using low-frequency ultrasound technology, systematically investigate the effects of ultrasonic parameters on particle size, stability, and encapsulation efficiency, and further evaluate the impact of this nanoemulsion treatment on quality indicators—including color, weight loss, firmness, soluble solids content, phenolic content, antioxidant enzyme activities, and microbial counts—of fresh-cut pitaya during refrigerated storage. Compared with chemical preservatives, taurine as a natural amino acid offers inherent safety advantages for food applications. This work provides a theoretical basis and technical support for the efficient utilization of taurine and the development of preservation technologies for fresh-cut pitaya.

## 2 Materials and methods

### 2.1 Materials and reagents

Fresh pitaya (red flesh variety, Red Dragon No. 1) was purchased from a local orchard in Jinzhou, China. Fruits of uniform maturity that were free of mechanical damage, pests, and visible disease were selected and pre-cooled at 4 °C before use.

Chitosan (deacetylation degree ≥90%), tea polyphenols (purity ≥98%), clove essential oil (food grade), taurine (purity ≥99%), glacial acetic acid (analytical grade), glycerol (analytical grade), Tween-80, Span-80, and food-grade soybean oil were purchased from Sinopharm Group Chemical Reagents Co., Ltd. (Shanghai, China). Folin–Ciocalteu reagent, gallic acid standard (purity ≥99%), catechol, resorcinol, phosphate-buffered saline (PBS), trichloroacetic acid (TCA), thiobarbituric acid (TBA), and sodium hydroxide (NaOH, analytical grade) were obtained from Aladdin Reagents Co., Ltd. (Shanghai, China). Plate count agar (PCA) and rose bengal medium (for mold and yeast enumeration) were purchased from Beijing Land Bridge Technology Co., Ltd. (Beijing, China). Deionized water was used throughout the experiments. All other reagents were of analytical grade unless otherwise specified.

### 2.2 Preparation of sulfuric acid-containing Nanoemulsions

#### 2.2.1 Preparation of crude emulsion

An oil-in-water (O/W) nanoemulsion using a modified version of the method described by Wang et al. (15). Taurine was dissolved in deionized water to prepare aqueous solutions with mass fractions of 0.5, 1.0, and 1.5%. Soybean oil constituted 10% (v/v) of the total emulsion volume. Tween-80 and Span-80 were used as emulsifiers at a mass ratio of 3:1 to obtain a combined hydrophilic–lipophilic balance (HLB) value of 12, and the total emulsifier mass was equivalent to 20% of the oil phase mass. The aqueous phase was heated to 40 °C, and the oil phase containing emulsifiers was slowly added and mixed. The mixture was pre-mixed for 10 min using a magnetic stirrer (85–2, Sile Instrument Co., Ltd., Shanghai, China) at 800 rpm to obtain a crude emulsion.

#### 2.2.2 Low-frequency ultrasound-assisted emulsification

The crude emulsion was placed in a low-frequency ultrasonic cell disruption instrument (KQ-400 KDE ultrasonic instrument; Kunshan Ultrasonic Instrument Co., Ltd.). The ultrasonic frequency was fixed at 20 kHz, and the ultrasonic powers were 200 W, 400 W, and 600 W. Each sample was sonicated for 20 min while its temperature was maintained at 25 °C in an ice-water bath. After sonication, the emulsion was allowed to cool to 25 °C, and the resulting emulsion containing taurine was obtained. A blank emulsion without taurine served as a control.

### 2.3 Characterization of nanoemulsions

#### 2.3.1 Particle size and Polydispersity index (PDI)

Freshly prepared nanoemulsions were diluted with deionized water at a ratio of 1:40 (v/v) to minimize multiple scattering effects. Preliminary dilution experiments confirmed that the ratio suitable for measurement was 1:40. The average particle size and PDI were measured using a Zetasizer Nano ZS90 laser particle size analyzer (Malvern, United Kingdom). Each sample was measured three times, and the results were expressed as “average value ± standard deviation.”

#### 2.3.2 Zeta potential

The zeta potential of each nanoemulsion was measured at 25 °C using the same Zetasizer Nano ZS90 instrument. Each sample was measured in triplicate, and the results are expressed as mean ± standard deviation.

#### 2.3.3 Encapsulation rate and drug loading capacity

Nanoemulsions containing 0.5, 1.0, and 1.5% taurine were prepared using 400 W ultrasonic power at 20 kHz for 20 min. A 1.0-mL aliquot of each formulation was centrifuged (Hunan Herexi Instrument Equipment Co., Ltd., Changsha, China) at 12,000 rpm at 4 °C for 30 min. The lower aqueous phase was carefully collected, filtered through a 0.22-μm polyethersulfone (PES) membrane filter (Millipore, Burlington, MA, United States), and analyzed for free taurine content using high-performance liquid chromatography (HPLC). HPLC analysis was performed on an Agilent 1,260 Infinity system (Agilent Technologies, Santa Clara, CA, United States) equipped with a C18 column (4.6 mm × 250 mm, 5 μm, Agilent ZORBAX SB-C18), with a methanol-phosphate buffer (pH 7.0, 30:70, v/v) mobile phase at a flow rate of 1.0 mL/min, column temperature of 30 °C, and detection wavelength of 360 nm (oxalyl aldehyde pre-drying method).

Encapsulation efficiency (EE%) was calculated as follows:


$$\begin{array}{l} \mathrm{EE}\%=(\text{total taurine added amount}—\text{free taurine amount})\\ /\text{total taurine added amount}\times 100\% \end{array}$$ (1)

Drug loading (DL) was calculated as follows:


$$\mathrm{DD}=(\begin{array}{l} \text{total taurine added amount}\\ —\text{free taurine amount} \end{array})/\text{emulsion mass}$$ (2)

Each sample was measured in triplicate, with the results expressed as the mean ± standard deviation.

#### 2.3.4 Stability evaluation

Nanoemulsions prepared under the optimized conditions were stored in light-protected amber glass vials manufactured by CNW Technologies, Shanghai, China, at 4 or 25 °C. Samples were evaluated on days 0, 7, 14, 21, and 28 for changes in particle size, polydispersity index, zeta potential, phase separation, precipitation, and color (16).

#### 2.3.5 Microscopic morphology observation

The microscopic morphology of the taurine-loaded nanoemulsion (T-NE) during storage was observed using an optical microscope (Nexcope NE910, Ningbo Yongxin Optics Co., Ltd., China) and a label-free live cell imaging system (SC3000, Zircon Optoelectronics, China). T-NE samples freshly prepared (day 0) and stored at 4 °C and 25 °C for 28 days were appropriately diluted with deionized water. An aliquot of 10 μL was placed on a glass slide, covered with a coverslip, and observed under the optical microscope at 40 × magnification and under the SC3000 system at 60 × magnification, respectively. The SC3000 system is based on non-interferometric intensity diffraction tomography (IDT) technology, which utilizes the refractive index contrast of the sample as an endogenous imaging contrast mechanism, enabling high-resolution imaging without any fluorescent labeling.

### 2.4 Processing and grouping of fresh-cut pitaya

#### 2.4.1 Sample preparation

After pre-cooling, the fruits were rinsed with deionized water and peeled manually. The flesh was cut with sterile stainless-steel knives into fan-shaped slices approximately 1 cm thick and 3 cm in diameter, with each slice weighing approximately 5 g. 2.4.2 Experimental Grouping.

The following four treatment groups were set up:

- *Control group (CK)*: Freshly cut pitaya slices were immersed in deionized water for 2 min.
- *T group (free taurine group)*: The slices were immersed in 1.0% taurine aqueous solution (w/v) for 2 min.
- *NE group (blank nanoemulsion group)*: The slices were immersed in a nanoemulsion without taurine for 2 min.
- *T-NE group (taurine-containing nanoemulsion group)*: The slices were immersed in a nanoemulsion containing 1.0% taurine prepared under optimal conditions for 2 min.

Each treatment comprised three independent replicates, and 10 slices of pitaya were used for each replicate. After immersion, the fruit slices were placed on sterile filter paper to drain the surface liquid and then placed in polypropylene preservation boxes with breathable holes. Each box contained 10 slices with a total mass of approximately 50 g. The samples were stored at 4 °C and 85 ± 5% relative humidity and were analyzed on days 0, 2, 4, 6, and 8.

### 2.5 Evaluation indicators for preservation effect

#### 2.5.1 Color (L*, a*, b* values)

The brightness (L*), red-green (a*), and yellow-blue (b*) values of the fruit surfaces were measured using a CR-400 colorimeter (Konica Minolta, Japan). Three points were randomly selected from each sample for measurement, and the degree of color change was represented by the total color difference value ΔE.


$$\mathrm{\Delta E}=\sqrt{\;{\left(\Delta L^{*}\right)}^{2}+{\left(\Delta a^{*}\right)}^{2}+{\left(\Delta b^{*}\right)}^{2}}$$ (3)

#### 2.5.2 Weight loss rate

The fruit slices in each replicate were weighed before and after storage using an FA1204C analytical balance with a readability of 0.1 mg, manufactured by Shanghai Yueping Scientific Instrument Co., Ltd., Shanghai, China. Weight loss was calculated using the following equation:


$$\begin{array}{l} \text{Weight loss rate}\;(\%)=(\begin{array}{l} \text{initial mass}—\text{post}\\ -\text{storage mass} \end{array})\\ /\text{initial mass}\times 100 \end{array}$$ (4)

#### 2.5.3 Firmness

A puncture test was conducted using a TA-XT Plus texture analyzer (Stable Micro Systems, United Kingdom). The probe diameter was 2 mm, the test speed was 1 mm/s, and the puncture depth was 5 mm. The maximum force (N) was recorded as the firmness value. Each sample was tested six times.

#### 2.5.4 Total soluble solid content (TSS)

Pitaya pulp was homogenized and centrifuged at 4,000 rpm for 10 min (Hunan Herexi Instrument Equipment Co., Ltd., Changsha, China), and the supernatant was collected. The soluble solids content (%) was measured using a handheld refractometer (Atago, Japan).

#### 2.5.5 Total phenolic content (TPC)

The total phenolic content was determined using the Folin–Ciocalteu method (17). Fruit pulp (2 g) was added to 10 mL of 70% methanol and homogenized at 4 °C in an ice bath, followed by centrifugation (Hunan Herexi Instrument Equipment Co., Ltd., Changsha, China) at 10,000 rpm for 15 min at 4 °C. The supernatant was reacted with Folin–Ciocalteu reagent (Beijing Solarbio Science and Technology Co., Ltd., Beijing, China), and absorbance was measured at 765 nm using a UV-2600 spectrophotometer (Shimadzu Corporation, Kyoto, Japan). A standard curve was constructed using gallic acid as the standard. Results are expressed as milligrams of gallic acid equivalent per 100 g fresh weight (mg GAE/100 g FW), and each sample was analyzed in triplicate.

#### 2.5.6 DPPH radical scavenging rate determination

Pitaya pulp weighing 2.0 g was mixed with 10 mL of 80% methanol, homogenized in an ice bath, and centrifuged (Hunan Herexi Instrument Equipment Co., Ltd., Changsha, China) at 10,000 rpm at 4 °C for 15 min. The supernatant was used as the test sample. An aliquot (2.0 mL) of the sample solution was mixed with 2.0 mL of 0.1 mmol/L DPPH methanol solution (freshly prepared with anhydrous methanol). The absorbance at 517 nm was measured (Asample). The background absorbance of the sample (Ablank) was measured using anhydrous methanol instead of the DPPH solution, and the blank absorbance of DPPH (Acontrol) was measured using anhydrous methanol instead of the sample. The DPPH free radical scavenging rate was calculated using the following formula:


$$\text{Scavenging rate}\;(\%)=[\begin{array}{l} 1—(\text{Asample}-\text{Ablank})\\ /\text{Acontrol} \end{array}]\times 100$$ (5)

Each sample was measured in triplicate, and the results were expressed as the average value ± standard deviation.

#### 2.5.7 Antioxidant enzyme activity

##### 2.5.7.1 Superoxide dismutase (SOD) activity

SOD activity was determined using the nitroblue tetrazolium (NBT) photoreduction method (18). The enzyme activity unit was defined as the amount of enzyme required to inhibit NBT photoreduction by 50% as one activity unit (U), and the results were expressed in U/g FW.

##### 2.5.7.2 Peroxidase (POD) activity

POD activity was determined using the crotonic acid method (19). One enzyme activity unit (U) was defined as the increase in absorbance at 470 nm by 0.01 per minute, and the results were expressed as U/g FW.

##### 2.5.7.3 Catalase (CAT) activity

CAT activity was determined using the ultraviolet absorption method (1). One enzyme activity unit (U) was defined as the decrease in absorbance at 240 nm by 0.01 per minute, and the results were expressed as U/g FW.

#### 2.5.8 Malondialdehyde (MDA) content

MDA content was determined using the thiobarbituric acid (TBA) method (20). The supernatant of the homogenate was reacted with the TBA reagent, and the absorbance was measured at 532 and 600 nm. The results were expressed in nmol/g FW.

#### 2.5.9 Microbial Indicator determination

Total viable counts and mold and yeast counts were determined according to Fan et al. (21). A 10.0-g fruit sample was homogenized for 2 min with 90 mL of sterile 0.85% sodium chloride solution to prepare a 10-fold dilution. Serial 10-fold dilutions were then prepared, and 1.0 mL of an appropriate dilution was plated on plate count agar and rose bengal medium. Plate count agar plates were incubated at 36 ± 1 °C for 48 h to enumerate total viable microorganisms. Rose bengal plates were incubated at 28 ± 1 °C for 5 days to enumerate molds and yeasts. Three replicate plates were analyzed for each dilution, and counts are expressed as log CFU/g.

### 2.6 Data processing and analysis

All experiments were independently repeated three times, and the results are presented as the mean ± standard deviation (SD). Two-way analysis of variance was performed using SPSS version 26.0, with treatment and storage time as fixed factors (IBM Corp., Armonk, NY, United States). When a significant interaction between treatment and storage time was detected, treatment means were compared at each time point using one-way analysis of variance followed by Duncan’s multiple-range test. Statistical significance was defined as *p* < 0.05. Graphs were drawn using Origin 2021 (OriginLab Corp., Northampton, MA, United States).

## 3 Results and discussion

### 3.1 Effect of ultrasonic power on particle size and zeta potential of nanoemulsions

Ultrasonic power significantly affected the particle size, distribution, and zeta potential of the nanoparticles (Table 1). The untreated (0 W) crude emulsion contained micrometer-scale droplets, with a D50 of 2891.33 nm, a polydispersity index (PDI) of 0.73, and an absolute Zeta potential of only −10.86 mV, indicating poor colloidal stability. After applying 200 W of ultrasonic power, the cavitation effect broke up the droplets, reducing the D50 to 175.43 nm, PDI to 0.28, and absolute Zeta potential to −28.67 mV (*p* < 0.05), significantly improving the emulsification effect. Increasing the power to 400 W further reduced the D50–112.58 nm and the polydispersity index to 0.17 and produced a zeta potential of −35.84 mV, indicating a relatively uniform and stable dispersion. At 600 W, the D50, polydispersity index, and zeta potential were 109.37 nm, 0.16, and −36.21 mV, respectively, and did not differ significantly from the corresponding values obtained at 400 W at *p* > 0.05. These findings indicate that the emulsification response reached a plateau at 400 W. Because higher power did not provide a meaningful improvement and could increase heat generation and energy consumption, 400 W was selected as the optimal ultrasonic power. A treatment time of 20 min and frequency of 20 kHz were selected based on preliminary optimization experiments.

**Table 1:** Effect of ultrasonic power on particle size and potential of nanoparticles.

Ultrasound power (*W*)	D10 (nm)	D50 (nm)	D90 (nm)	PDI	Zeta potential (mV)
0	1063.52 ± 42.18 a	2891.33 ± 87.56 a	6372.91 ± 145.23 a	0.73 ± 0.04 a	−10.86 ± 1.24 a
200	83.16 ± 2.45 b	175.43 ± 4.28 b	315.29 ± 6.71 b	0.28 ± 0.02 b	−28.67 ± 1.53b
400	70.23 ± 1.57 c	112.58 ± 2.13 c	161.42 ± 3.85 c	0.17 ± 0.01 c	−35.84 ± 1.26 c
600	68.45 ± 1.83 c	109.37 ± 2.46 c	157.93 ± 4.02 c	0.16 ± 0.02 c	−36.21 ± 1.09 c

Different letters represent different levels of significance. *p* < 0.05.

### 3.2 Effect of taurine concentration on encapsulation rate and drug loading

**Figure 1:**
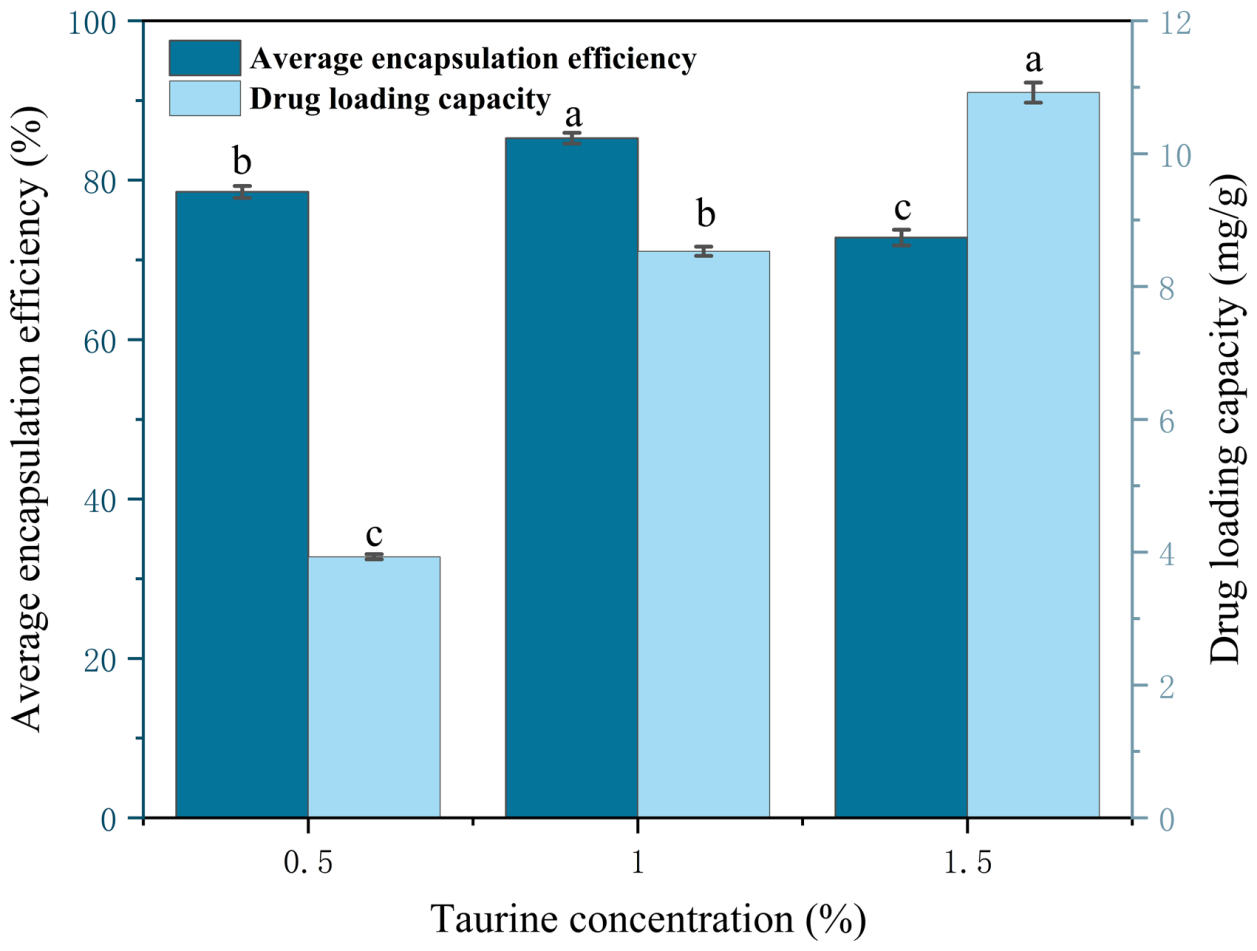
Effect of Taurine concentration on encapsulation rate and drug loading. Different letters represent different levels of significance. *p* < 0.05.

Under the optimal ultrasonic power of 400 W, the effects of different taurine concentrations (0.5, 1.0, and 1.5%) on the encapsulation efficiency (EE) and drug loading (DL) were investigated (Figure 1). Encapsulation efficiency increased from 78.53% at 0.5% taurine to 85.27% at 1.0% taurine and then decreased to 72.80% at 1.5% taurine, with significant differences between concentrations (*p* < 0.05). This trend can be explained as follows: at low concentrations, the emulsifier was relatively sufficient to effectively encapsulate taurine; at 1.0%, the emulsifier and taurine were optimally matched, and the interfacial membrane remained intact. At 1.5%, the viscosity of the aqueous phase increased; taurine competed for adsorption and interfered with emulsifier arrangement; at the same time, the particle size increased, reducing the interface area, resulting in decreased EE (22, 23). Unlike EE, DL continuously increased from 3.93 mg/g at 0.5% to 8.53 mg/g at 1.0%, and to 10.92 mg/g at 1.5% (*p* < 0.05). Although the EE decreased at the highest concentration, the total taurine added increased substantially, and the absolute mass of the encapsulated taurine remained higher; therefore, the DL increased. Considering particle size, polydispersity index, zeta potential, and encapsulation efficiency, 1.0% taurine was selected as the optimal concentration.

### 3.3 Storage stability of nanoemulsions

**Figure 2:**
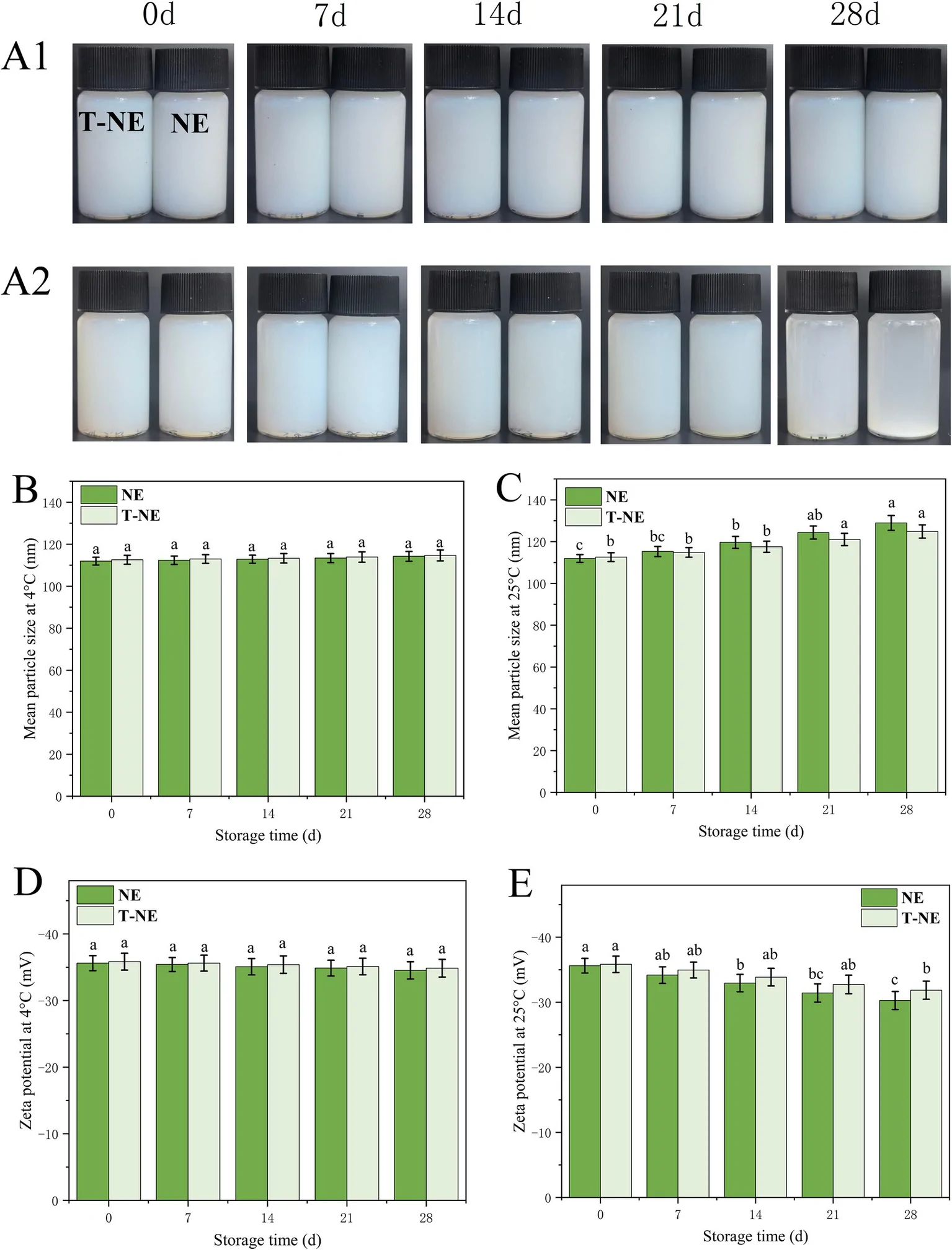
Storage stability of taurine-loaded nanoemulsions. **(A)** Appearance of nanoemulsions stored at 4 °C **(A1)** and 25 °C **(A2)** over 28 days. **(B,C)** mean particle size; **(D,E)** zeta potential. CK, Control; T, Free taurine; NE, Blank nanoemulsion; T-NE, Taurine-loaded nanoemulsion. Different letters represent different levels of significance. *p* < 0.05.

The particle size, polydispersity index, zeta potential, and visual appearance of the blank nanoemulsion and taurine-loaded nanoemulsion were monitored for 28 days at 4 and 25 °C under the optimized preparation conditions of 400 W and 1.0% taurine (Figure 2). At 4 °C, the particle sizes of both NE and T-NE did not show significant changes (*p* > 0.05), gradually increasing from 111.94 nm and 112.58 nm to 114.22 nm and 114.65 nm, respectively. The PDI remained between 0.16 and 0.19, and the absolute value of zeta potential was >34 mV, with no significant decrease (*p* > 0.05). Neither group showed stratification, precipitation, or discoloration, indicating that the stability of the emulsions was maintained for a long time at 4 °C.

At 25 °C, the particle sizes of both groups significantly increased over time (*p* < 0.05) starting from 7 days. At 28 days, the particle size of NE increased to 128.96 nm, and that of T-NE increased to 124.86 nm; the particle size of T-NE was slightly smaller than that of NE at each time point. This indicated that taurine could inhibit droplet aggregation and Ostwald ripening. In terms of PDI, NE significantly increased to 0.20 and 0.21 at 21 days and 28 days, respectively, while T-NE only increased to 0.21 at 28 days. The deterioration lagged. The absolute value of zeta potential decreased at 25 °C: NE significantly decreased from the 14th day (−32.95 mV), and dropped to −30.27 mV at 28 days; T-NE significantly decreased at 14 and 21 days (−33.86 and −32.74 mV), but remained at −31.85 mV at 28 days, slightly higher than NE. The absolute values of the potentials in both groups remained >30 mV, indicating moderate stability. In terms of appearance, NE showed very slight water precipitation at 28 d, whereas T-NE showed no significant stratification.

Both formulations therefore showed good storage stability at 4 °C. Although gradual destabilization occurred at 25 °C, the taurine-loaded nanoemulsion remained slightly more stable than the blank formulation. These findings support refrigerated storage of the formulation before its use in fresh-cut pitaya preservation (≤7 days).

### 3.4 Microscopic morphology of nanoemulsions during storage

**Figure 3:**
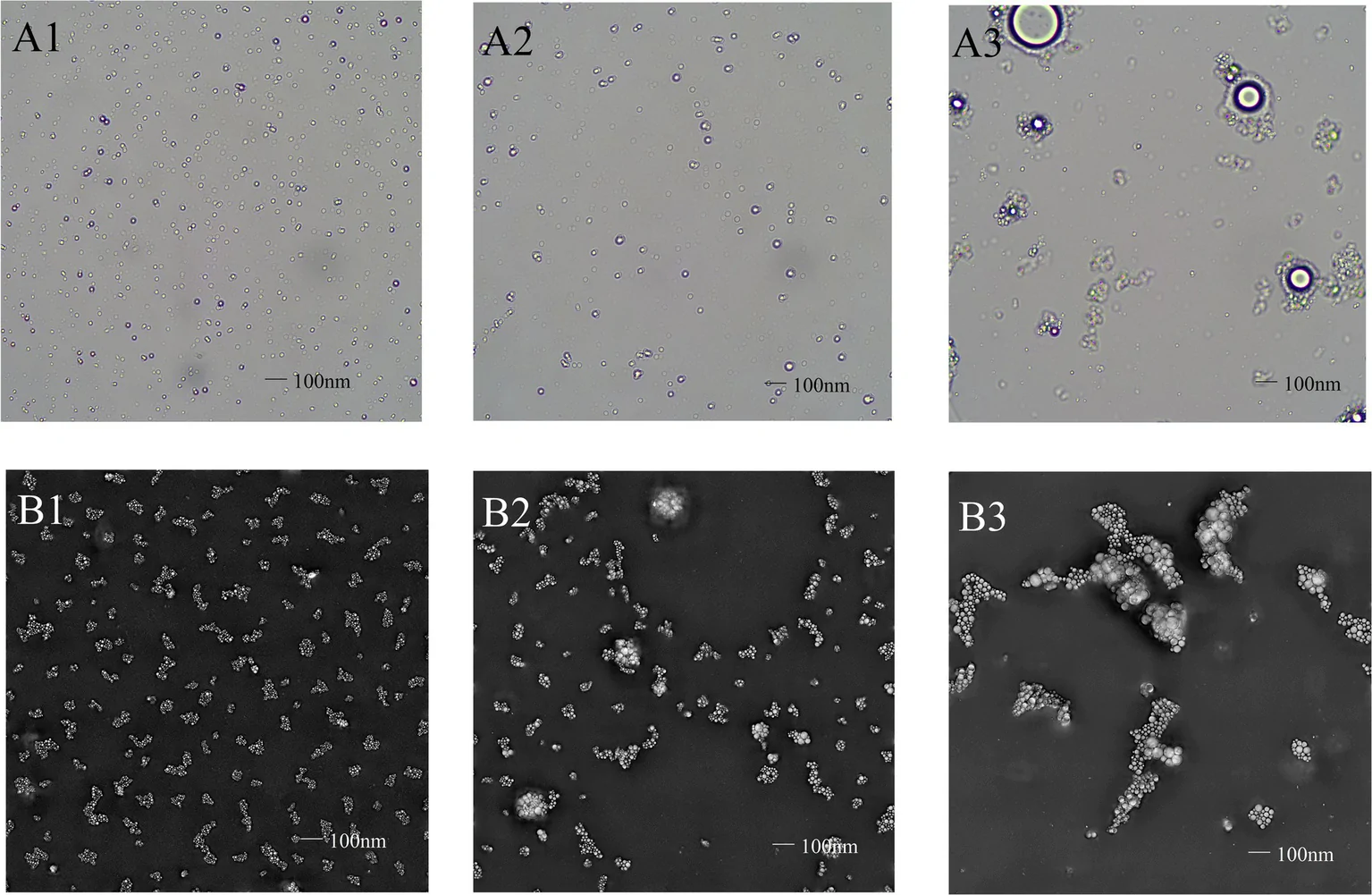
Microscopic morphology of taurine-loaded nanoemulsion (T-NE) during storage. **(A)** Optical microscopy images (NE910, 400 × magnification): **(A1)** freshly prepared (day 0); **(A2)** stored at 4 °C for 28 days; **(A3)** stored at 25 °C for 28 days. **(B)** Label-free live cell imaging system images (SC3000, 600 × magnification): **(B1)** freshly prepared (day 0); **(B2)** stored at 4 °C for 28 days; **(B3)** stored at 25 °C for 28 days.

Microscopy was used to qualitatively examine changes in the morphology of the taurine-loaded nanoemulsion during storage (Figure 3). The freshly prepared formulation exhibited uniformly dispersed spherical droplets with small dimensions and no signs of aggregation, confirming excellent monodispersity. After 28 days at 4 °C, the emulsion remained largely dispersed, though a few small clusters appeared, indicating slight uniformity loss. These observations were consistent with the limited changes in particle size and zeta potential measured at 4 °C (24).

After 28 days at 25 °C, more pronounced droplet clustering and apparent coalescence were observed. Elevated temperature intensifies Brownian movement and droplet collisions; concurrently, diminished surface charge weakens inter-droplet repulsion, lowering the energy barrier against aggregation. Enhanced molecular mobility also accelerates broadening of the size distribution and compromises structural integrity. These microscopic findings are fully consistent with particle size and zeta potential measurements. Therefore, refrigeration at 4 °C is essential for preserving the nanoemulsion’s colloidal structure over extended storage, whereas ambient temperature accelerates irreversible destabilization. For practical applications in fresh-cut fruit preservation, the taurine-loaded nanoemulsion should be stored and transported under chilled conditions to ensure its functional performance and shelf-life.

### 3.5 Effects of different treatments on the color of fresh-cut pitaya

**Figure 4:**
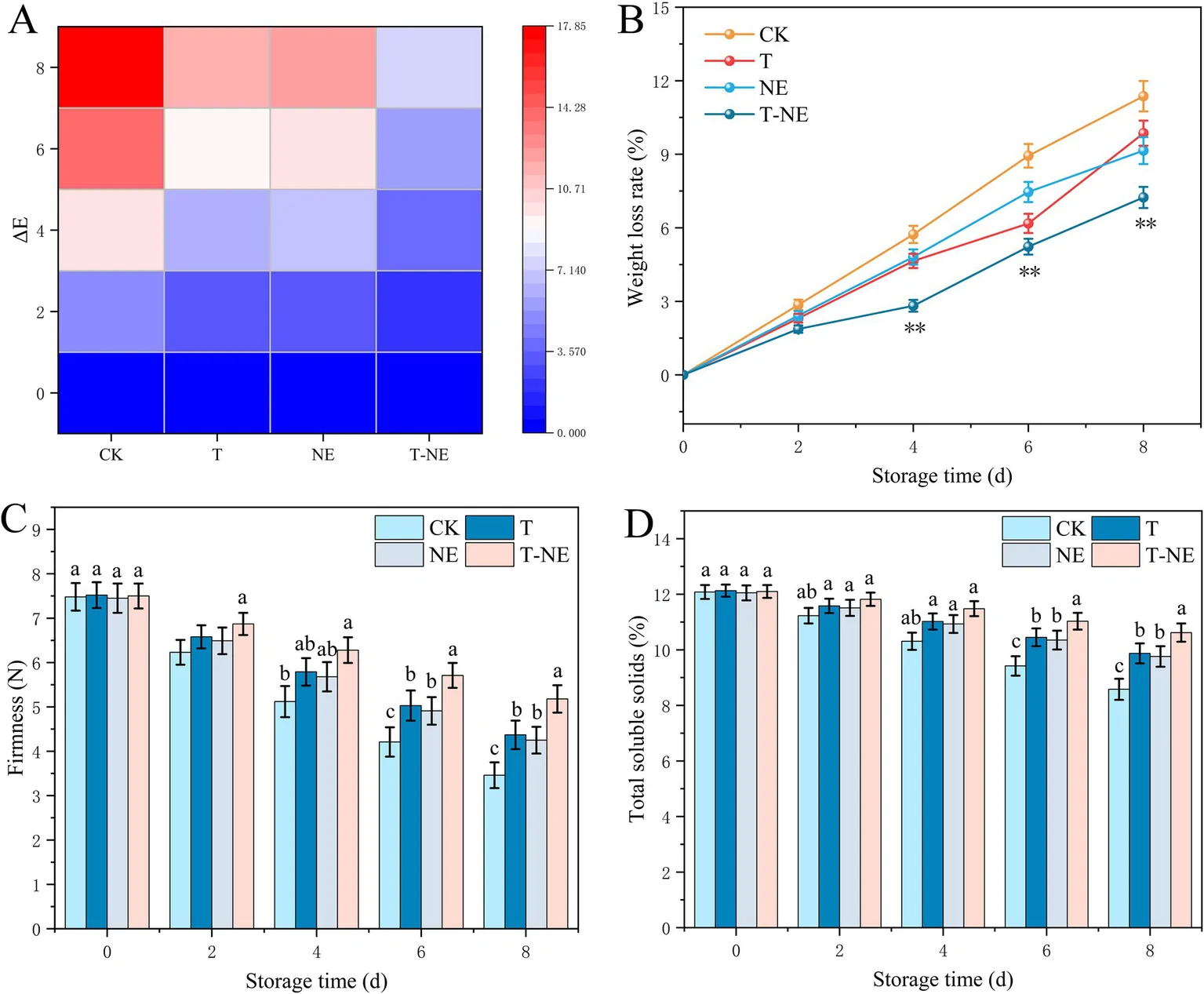
**(A)** Effects of different treatments on the color of fresh-cut pitaya. **(B)** Weight loss; **(C)** Firmness; **(D)** Total soluble solids. CK, Control; T, Free taurine; NE, Blank nanoemulsion; T-NE, Taurine-loaded nanoemulsion. Different letters represent different levels of significance. *p* < 0.05.

During storage, the L* and a* values of all treatment groups decreased, while b* and total color difference (ΔE) increased. However, the extent of change varied among treatments (Figure 4A). The control group deteriorated the fastest: at day 8, L* dropped from 72.45 to 56.42 (22.1% decrease), a* decreased from 28.36 to 14.68 (loss of nearly 50%), b* increased to 8.73, and ΔE reached 17.85. The effects of free taurine (T) and blank nanoemulsion (NE) were similar, with L* approximately 62.5, a* approximately 19.1, and ΔE approximately 11.9 at day 8, all significantly better than control (*p* < 0.05), whereas no significant difference was observed between T and NE. In contrast, the taurine-loaded nanoemulsion (T-NE) group had the best color protection: L* remained at 66.25, a* was 22.97, b* only rose to 6.67, ΔE was only 7.68, and all indicators were significantly better for T-NE than for the other three groups (*p* < 0.05). The superior performance of T-NE can be attributed to a synergistic mechanism: the nanoemulsion matrix forms a physical barrier that reduces oxygen contact, while simultaneously enhancing the adhesion and penetration of taurine onto the fruit surface, thereby more effectively inhibiting enzymatic browning and oxidative discoloration (13, 25). This combined effect is consistent with previous reports that nanoemulsion-based coatings can effectively delay color deterioration in fresh-cut fruits (9, 14). Therefore, T-NE treatment most effectively maintained the commercial color of fresh-cut pitaya during refrigerated storage.

### 3.6 Effects of different treatments on the weight loss rate of fresh-cut pitaya

The rate of weight loss increased with storage time, with the control group showing the fastest growth rate (Figure 4B). On the 8th day, the weight loss rate of the control group reached 12.37% and the fruit became significantly wilted; the weight loss rate in the free taurine (T) and blank nanoemulsion (NE) groups were 9.86 and 10.15%, respectively, significantly lower than that of the control group (*p* < 0.05); however, T and NE showed no difference. The T-NE group exhibited the lowest weight loss rate of 8.24%, which was significantly lower than that of the other groups (*p* < 0.05). The improved water retention in T-NE-treated samples can be attributed to a dual mechanism: the nanoemulsion matrix forms a physical barrier that reduces surface water evaporation, while taurine, as an osmotic regulator, helps maintain cell turgor and reduces intracellular water leakage. The sustained release of taurine from the nanoemulsion droplets likely prolongs this protective effect. Therefore, T-NE treatment effectively delayed the wilting of fresh-cut pitaya during refrigerated storage.

### 3.7 Effects of different treatments on the firmness and soluble solid content of fresh-cut pitaya

Firmness and TSS content reflect the textural and nutritional quality of fruits, respectively. During storage, both firmness and TSS decreased in all treatment groups, although the extent of these reductions differed among treatments (Figures 4C,D). The initial values showed no significant differences among the groups. On the 8th day, the firmness of the control group dropped to 3.46 N (loss of 53.7%), and TSS dropped to 8.58 °Brix (loss of 29.0%). The free taurine and blank nanoemulsion treatments produced similar effects, with firmness approximately 4.3 N (loss of 42%) and TSS approximately 9.8 °Brix (loss of 19%), both significantly better than control (*p* < 0.05); the firmness of the taurine-containing nanoemulsion group (T-NE) was 5.18 N (loss of 30.9%), and TSS was 10.62 °Brix (loss of 12.2%), significantly better than other groups (*p* < 0.05). This is because nanoemulsions form a physical barrier that reduces water evaporation and inhibits respiration metabolism, while taurine, an antioxidant and osmotic regulator, inhibits the activity of cell wall-degrading enzymes and stabilizes mitochondrial membrane function. T-NE maintains cell turgor and nutritional substrates most effectively through a synergistic release effect. Therefore, treatment with taurine-containing nanoemulsions can effectively delay the softening and nutritional deterioration of fresh-cut pitaya.

In summary, the NE matrix contributed primarily through a physical barrier that reduced water evaporation and oxygen permeation, while the T group exerted its effects mainly via antioxidant and osmotic regulation. The T-NE treatment combined both mechanisms through sustained release of taurine from the nanoemulsion droplets, thereby achieving the best preservation outcomes.

### 3.8 Effects of different treatments on total phenolic content

**Figure 5:**
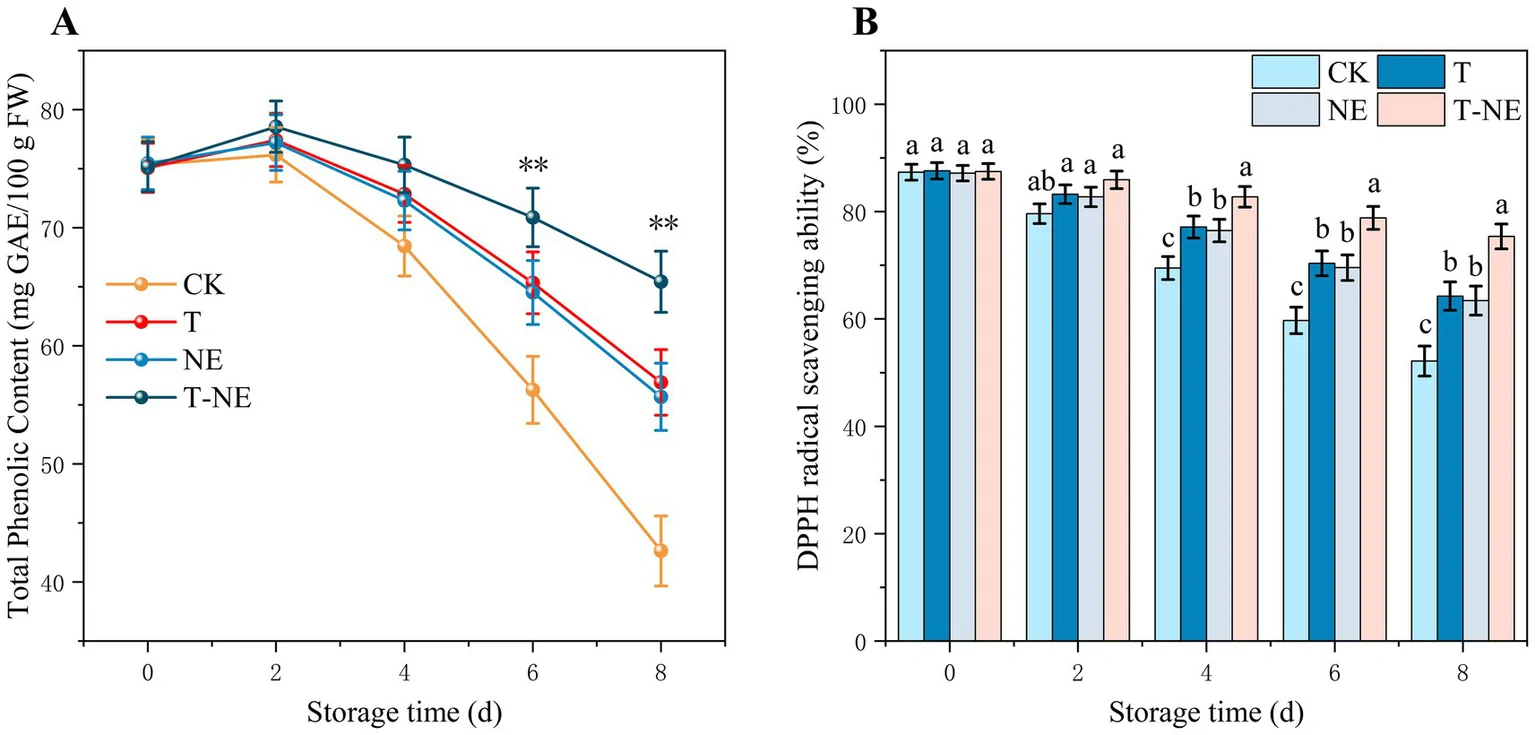
**(A)** Effects of different treatments on the total phenolic content of fresh-cut pitaya. **(B)** Effects of different treatments on the DPPH radical scavenging ability of fresh-cut pitaya. Different letters represent different levels of significance. *p* < 0.05. CK, control; T, free taurine; NE, blank nanoemulsion; T-NE, taurine-loaded nanoemulsion.

The total phenolic content initially increased and subsequently decreased during storage, although the magnitude of these changes varied among the treatment groups (Figure 5A). The initial values among all groups showed no significant difference. On day 2, the total phenolic content in the control group increased slightly to 76.2 mg/100 g, although this change was not statistically significant (*p* > 0.05). In contrast, the free taurine and blank nanoemulsion groups showed significant increases to 77.4 and 77.2 mg/100 g, respectively. These values did not differ significantly from each other but were both significantly higher than that of the control group (*p* < 0.05). The taurine-loaded nanoemulsion group exhibited the highest total phenolic content at 78.6 mg/100 g.

From day 4 onwards, the total phenol content of the control group rapidly decreased and reached 42.6 mg/100 g by day 8 (a loss of 43.4%). The T and NE groups maintained total phenol contents of 56.9 and 55.7 mg/100 g, respectively (loss of approximately 25%), with no significant difference between them. In contrast, the T-NE group maintained total phenolic content of 65.4 mg/100 g (loss of only 13.0%), which was significantly higher than those of the other groups (*p* < 0.05). Therefore, treatment with taurine-containing nanoemulsions was most effective in maintaining the phenolic-associated antioxidant quality of fresh-cut pitaya.

### 3.9 Effects of different treatments on the DPPH radical scavenging rate

The DPPH scavenging activity decreased with storage time, but the rate of decline varied among treatments (Figure 5B). The initial values showed no significant difference among the groups (87.2–87.6%). At 2 days of storage, the DPPH scavenging rate in the control group dropped to 79.6%, with a loss of 8.8%, while the DPPH scavenging rate in the free taurine (T) and blank nanoemulsion (NE) groups decreased to 83.2 and 82.8% respectively, both significantly higher than that in the control (*p* < 0.05), but the two showed no significant difference. The T-NE group had a scavenging rate of 85.9%, significantly higher than the other groups. By the 8th day, the rate in the control was 52.2% (loss of 40.3%), while that in the T and NE groups was approximately 63.8% (loss of 27%), whereas it was 75.4% (loss of only 13.8%) in the T-NE group, with significant differences (*p* < 0.05). The enhanced DPPH scavenging activity in T-NE-treated samples can be attributed to a synergistic mechanism. The nanoemulsion matrix forms a physical barrier that slows the oxidation of phenolic compounds, while taurine directly scavenges free radicals and may upregulate endogenous antioxidant enzymes. The sustained release of taurine from the nanoemulsion droplets likely prolongs this protective effect, thereby most effectively maintaining the *in vitro* antioxidant activity of fresh-cut pitaya during refrigerated storage.

### 3.10 Effects of different treatments on the activities of antioxidant enzymes (SOD, CAT) and Browning-related enzymes (PPO)

**Figure 6:**
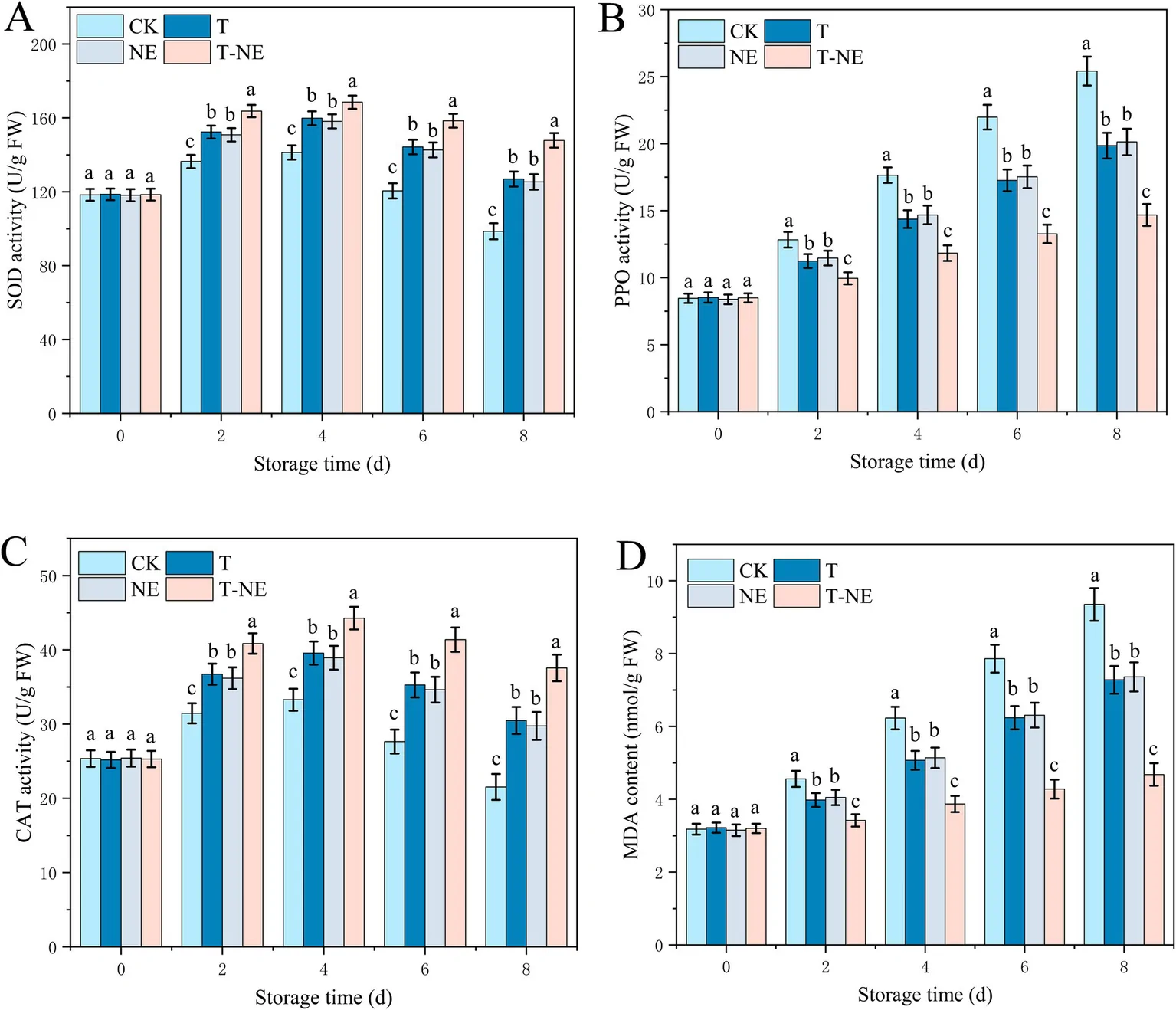
**(A)** Effects of different treatments on the SOD activity of fresh-cut pitaya; **(B)** Effects of different treatments on the PPO activity of fresh-cut pitaya; **(C)** Effects of different treatments on the CAT activity of fresh-cut pitaya; **(D)** Effects of different treatments on the MDA content of fresh-cut pitaya. Different letters represent different levels of significance. *p* < 0.05. CK, control; T, free taurine; NE, blank nanoemulsion; T-NE, taurine-loaded nanoemulsion.

SOD and CAT are the key antioxidant enzymes that remove reactive oxygen species, whereas PPO catalyzes enzymatic browning. During storage, the activities of SOD and CAT in each treatment group initially increased and then decreased, while PPO continued to increase, but the extent of change varied among treatments (Figure 6). Initial enzyme activity showed no significant difference among the groups.

Cutting stress induces an antioxidant defense response. At 2 days of storage, the SOD and CAT in the control group increased to 136.4 U/g FW and 31.5 U/g FW (by 15 and 24%), respectively. The free taurine (T) and blank nanoemulsion (NE) treatments significantly increased the activities of both enzymes, with no significant difference between the two. The T-NE group had the highest SOD and CAT activities (163.7 and 40.9 U/g FW), indicating that T-NE synergistically enhanced the stress-induced effect. Meanwhile, the PPO activity in the control group rapidly increased (12.8 U/g FW at 2 days, increasing by 51.7%), while the T and NE groups showed significantly lower activities than the control, with the T-NE group showing an activity of 9.95 U/g FW, suggesting that both taurine and nanoemulsions could inhibit the activation of browning enzymes.

After 4 days of storage, all enzymes reached their peak or continued to increase. The peak SOD activity was 141.3 U/g FW, and CAT was 33.3 U/g FW in the control group, followed by a decrease. The peak values in the T and NE groups were higher, while the T-NE group had the highest peak. During the same period, the PPO in the control group increased to 17.7 U/g FW (an increase of 108.6%), whereas that in the T-NE group was 11.8 U/g FW. In the following days (6–8 days), the SOD and CAT activities in the control rapidly decreased to below the initial level, indicating severe damage to the antioxidant system. The T and NE groups remained above the initial level, while the T-NE group showed SOD and CAT activities of 147.9 and 37.6 U/g FW (increases of 24.8 and 48.6%, respectively, compared to the initial level). On the 8th day, the PPO in the control reached 25.4 U/g FW, with severe browning; the T-NE group was 14.7 U/g FW (an increase of 73%), significantly inhibiting browning. These results suggest that taurine directly scavenges ROS and maintains antioxidant enzyme activity by protecting enzyme proteins from oxidative damage, while the nanoemulsion matrix provides sustained taurine release and a physical barrier against oxygen.

### 3.11 Effects of different treatments on malondialdehyde (MDA) content in fresh-cut pitaya

Malondialdehyde (MDA) is the final product of membrane lipid peroxidation and reflects the degree of cell membrane damage. During storage, MDA content in all treatment groups continuously increased, but the rate of increase varied significantly among treatments (Figure 6D). Initial values showed no significant differences among groups. At day 2 of storage, MDA in control increased to 4.56 nmol/g FW (an increase of 43.4%), whereas it was 3.98 and 4.05 nmol/g FW in T and NE, respectively, both significantly lower than control (*p* < 0.05), with no significant difference between the two. T-NE showed the lowest levels (3.42 nmol/g FW), suggesting that either taurine or nanoemulsion treatment alone can alleviate early lipid peroxidation, and that their combination exerts the greatest protective effect.

These differences continued to increase from days 4 to 8 of storage. On day 8, MDA in control reached 9.35 nmol/g FW, indicating severely damaged membrane structure. MDA was 7.28 and 7.36 nmol/g FW in T and NE, respectively, and only 4.68 nmol/g FW (an increase of 46.3%) in T-NE, significantly lower than the other groups (*p* < 0.05). The marked reduction in MDA accumulation in T-NE-treated samples can be explained by the combined effects of its components: the nanoemulsion matrix provides a physical barrier that limits oxygen diffusion to the fruit surface, thereby reducing the substrate for lipid peroxidation; simultaneously, taurine acts as a potent ROS scavenger and helps maintain the activity of antioxidant enzymes (SOD and CAT), as evidenced by the higher enzyme activities observed in the T-NE group. The sustained release of taurine from the nanoemulsion droplets likely prolongs this protective effect, resulting in the lowest MDA levels throughout storage. Therefore, taurine-loaded nanoemulsion effectively protected cell membrane integrity and delayed fruit senescence during refrigerated storage.

### 3.12 Effects of different treatments on the microbial growth of fresh-cut pitaya

**Figure 7:**
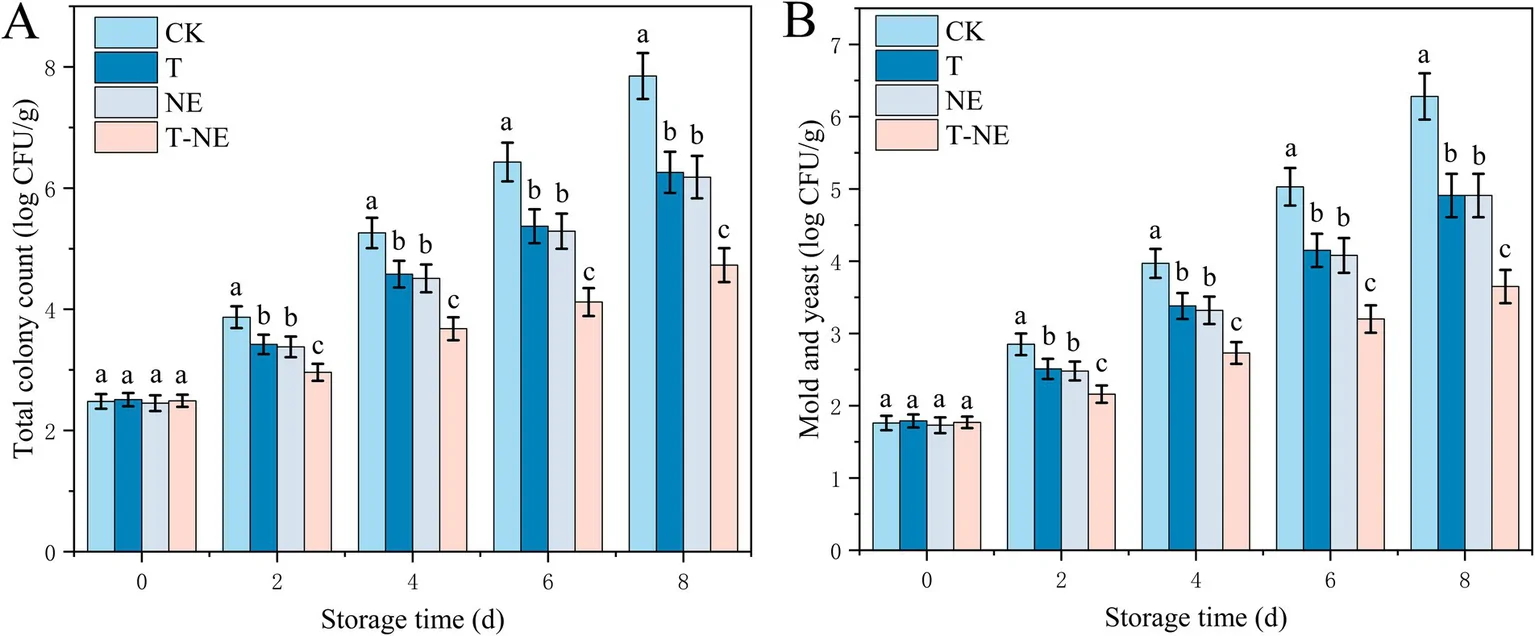
**(A)** Effects of different treatments on the total colony count of fresh-cut pitaya; **(B)** Effects of different treatments on the mold and yeast of fresh-cut pitaya. Different letters represent different levels of significance. *p* < 0.05. CK, control; T, free taurine; NE, blank nanoemulsion; T-NE, taurine-loaded nanoemulsion.

Total viable counts and mold and yeast counts are key indicators for evaluating the hygiene quality and shelf life of fresh-cut fruits and vegetables. During storage, microbial counts increased in all treatment groups, but the extent of this increase varied significantly depending on the treatment method (Figure 7).

No significant differences were observed among the groups in the initial total viable counts or mold and yeast counts, indicating comparable initial microbial loads. On the 2nd day of storage, the total colony count of the control group increased to 3.87 log CFU/g, while that of the free taurine group (T) and the blank nanoemulsion group (NE) was 3.42 and 3.38 log CFU/g, respectively, with no significant difference between them; however, both were significantly lower than that of the control (*p* < 0.05). The total colony count of the T-NE group was only 2.96 log CFU/g, and the counts of molds and yeasts were the lowest. These findings indicate that taurine has certain antibacterial activity, which can damage the cell membrane of microorganisms or interfere with their metabolism. Tween-80 and Span-80 in the nanoemulsions also have surface activity and can damage the cell structure of microorganisms; however, the antibacterial effects of the two used alone are similar, and both are limited.

As the storage time increased (4–6 days), the differences in total colony counts of the groups further increased. On the 4th day, the total colony count of the control reached 5.26 log CFU/g, whereas that of the T and NE groups was approximately 4.5 log CFU/g, and that of the T-NE group was only 3.68 log CFU/g. On the 6th day, control was close to the spoilage threshold (6.43 log CFU/g), the T and NE groups were approximately 5.3 log CFU/g, and the T-NE group was still controlled at 4.12 log CFU/g. By the 8th day, the endpoint control was severely spoiled (total colony count 7.85, molds and yeasts 6.28 log CFU/g), while the T and NE groups were better than the control but still at relatively high levels (approximately 6.2 and 5.0 log CFU/g). The total colony count and molds and yeasts in the T-NE group were only 4.73 and 3.65 log CFU/g, respectively, which were significantly lower than those in the other three groups (*p* < 0.05), and within the acceptable range for fresh-cut produce. Therefore, T-NE effectively controls microbial contamination of fresh-cut pitaya and significantly extends its shelf life.

The T-NE treatment showed the strongest antibacterial effect because of the synergistic mechanism. The nanoemulsions form a physical barrier on the fruit surface, reducing the water activity and oxygen contact, creating an unfavorable microenvironment for microbial proliferation, and the nanoencapsulation enhances the attachment and sustained release ability of taurine on the fruit surface, allowing sustained antibacterial activity. Therefore, taurine-containing nanoemulsions effectively control microbial contamination of fresh-cut pitayas and significantly extend their shelf life.

## 4 Conclusion

In this study, a taurine-loaded nanoemulsion was successfully fabricated using low-frequency ultrasound and evaluated as a preservative treatment for fresh-cut pitaya. The optimized formulation contained 1.0% taurine and was prepared at 400 W and 20 kHz for 20 min. It showed good colloidal stability for 28 days at 4 °C and delayed color deterioration, weight loss, softening, loss of total soluble solids and phenolic compounds, oxidative damage, and microbial growth during 8 days of refrigerated storage.

The taurine-loaded nanoemulsion consistently performed better than free taurine or the blank nanoemulsion, suggesting that the formulation improved the retention or effectiveness of taurine on the fruit surface. However, the proposed mechanisms involving coating formation, sustained taurine release, reduced oxygen transfer, and direct antimicrobial activity require experimental validation. Future studies should evaluate release kinetics, coating properties, sensory acceptability, formulation safety, performance across fruit varieties, and process scalability before commercial application.

## Data Availability

The original contributions presented in the study are included in the article/supplementary material, further inquiries can be directed to the corresponding author.
